# Supplementary figures and images for: Telomere length and brain imaging phenotypes in UK Biobank
Source: PLoS One. 2023 Mar 22;18(3):e0282363. doi: 10.1371/journal.pone.0282363 (PMC10032499; doi:10.1371/journal.pone.0282363)

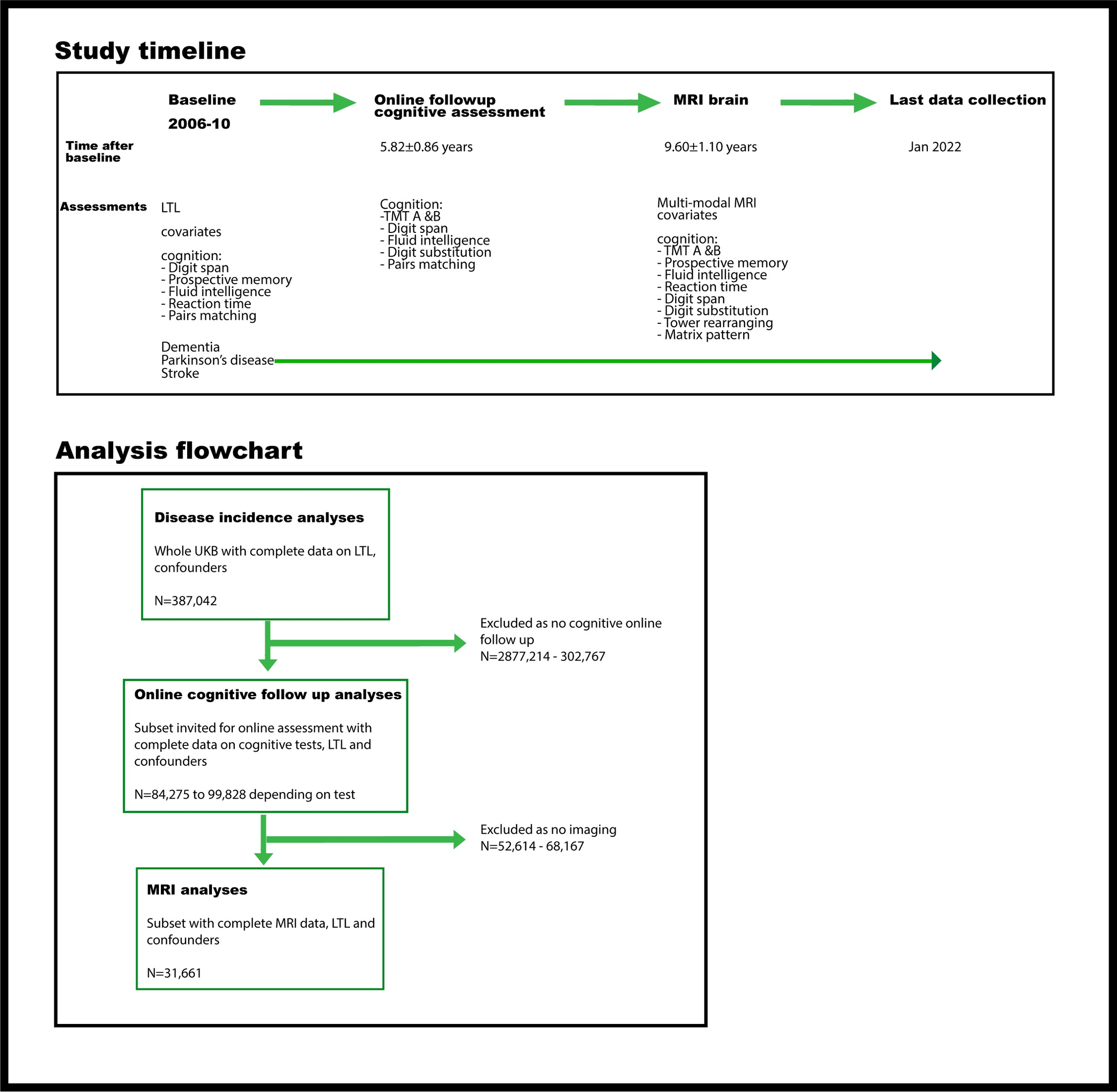

Supplement: S1 Fig — Abbreviations: LTL–leucocyte telomere length, MRI–magnetic resonance imaging, TMT–trail-making test. (TIF) [file pone.0282363.s001.tif]

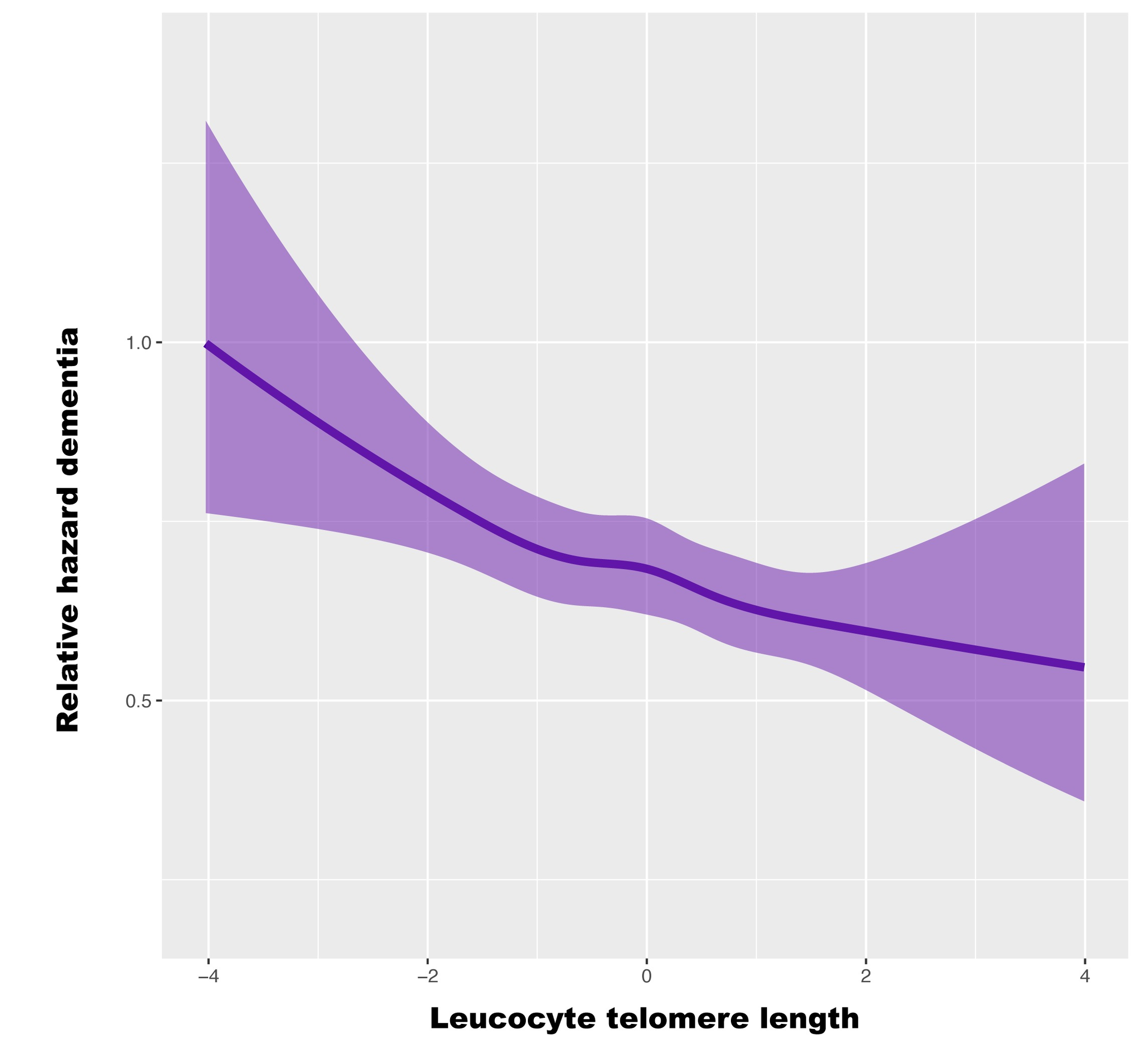

Supplement: S2 Fig — Hazards are plotted relative to that of median telomere length. Restricted cubic splines (5 knots, quintiles) are applied to quantile normalized LTL. Cox proportional hazards models adjusted for: age, age2, age3, sex, body mass index, educational qualifications, Townsend Deprivation Index, household income, historical job code, smoking, alcohol intake, leucocyte count, genetic ancestry. (TIF) [file pone.0282363.s002.tif]
