# Supplementary material for: Telomere length and brain imaging phenotypes in UK Biobank
Source: PLoS One. 2023 Mar 22;18(3):e0282363. doi: 10.1371/journal.pone.0282363 (PMC10032499; doi:10.1371/journal.pone.0282363)
Supplement: S1 File — (DOCX) [file pone.0282363.s009.docx]

**Cox proportional hazards assumptions checks**

# Dementia – after stratification for age quintile, BMI quintile and job

cox.zph(cox)

chisq df p

TL.qn 1.1363 1 0.286

sex 4.5392 1 0.033

qualifications 3.9751 6 0.680

TDI 0.2923 1 0.589

smoking 1.0335 2 0.596

alcohol 3.3084 1 0.069

income 9.1906 6 0.163

pca_1 0.5204 1 0.471

pca_2 0.2267 1 0.634

pca_3 1.2578 1 0.262

pca_4 0.0363 1 0.849

pca_5 0.1378 1 0.710

pca_6 1.6284 1 0.202

pca_7 0.7689 1 0.381

pca_8 0.6673 1 0.414

pca_9 0.8155 1 0.367

pca_10 0.0233 1 0.879

wbc 1.8984 1 0.168

GLOBAL 36.4081 29 0.162

# Parkinson’s disease – no stratification needed

cox.zph(cox)

chisq df p

TL.qn 1.69e-04 1 0.9896

age_ax 2.39e-04 1 0.9877

age2o 5.37e-01 1 0.4638

sex 5.57e-01 1 0.4555

qualifications 3.29e+00 6 0.7715

TDI 6.54e-01 1 0.4189

smoking 2.60e-01 2 0.8779

alcohol 7.41e+00 1 0.0065

BMI 3.89e+00 1 0.0487

income 4.45e+00 4 0.3485

job_1 2.35e-01 1 0.6277

job_2 6.61e-01 1 0.4162

job_3 2.68e-02 1 0.8700

job_4 9.67e-01 1 0.3253

pca_1 1.45e-01 1 0.7030

pca_2 2.10e+00 1 0.1472

pca_3 5.88e+00 1 0.0153

pca_4 2.69e-01 1 0.6043

pca_5 5.89e-01 1 0.4428

pca_6 3.59e-03 1 0.9522

pca_7 3.01e-02 1 0.8622

pca_8 4.14e-01 1 0.5199

pca_9 1.23e+00 1 0.2669

pca_10 4.26e+00 1 0.0390

wbc 1.99e-01 1 0.6554

GLOBAL 3.57e+01 34 0.3895

# CVA – after stratification for sex, job, and genetic ancestry principle component analysis component 9

cox.zph(cox)

chisq df p

TL.qn 0.0114 1 0.915

Age 0.6010 1 0.438

age2o 0.0356 1 0.850

qualifications 5.6959 6 0.458

TDI 2.6662 1 0.102

alcohol 0.6005 1 0.438

BMI 0.2074 1 0.649

Income 1.2421 4 0.871

pca_1 0.2471 1 0.619

pca_2 2.3248 1 0.127

pca_3 2.4088 1 0.121

pca_4 0.9552 1 0.328

pca_5 0.0175 1 0.895

pca_6 2.0192 1 0.155

pca_7 0.0103 1 0.919

pca_8 0.9141 1 0.339

pca_10 2.9085 1 0.088

wbc 0.6842 1 0.408

GLOBAL 23.8574 26 0.584

**Abbreviations:**

TL.qn – leucocyte telomere length, quantile normalized

TDI – Townsend Deprivation Index

BMI – Body mass index

PCA – principle components analysis

Wbc – white blood cell count (leucocytes)
