# Supplementary material for: Telomere length and brain imaging phenotypes in UK Biobank
Source: PLoS One. 2023 Mar 22;18(3):e0282363. doi: 10.1371/journal.pone.0282363 (PMC10032499; doi:10.1371/journal.pone.0282363)
Supplement: Attachment — Submitted filename: Reviewers rebuttal.docx [file pone.0282363.s010.docx]

**Reviewers rebuttal**

**Reviewer #1:** In this work association analyses between leucocyte telomere length (LTL) and brain multi-modal MRI traits were carried out based on the 31,661 UK Biobank participants. As the largest and richest study to date examining relationships between LTL and MRI markers of brain structure and function. this work includes many interesting conclusions and findings. I only have the following questions.

1) For the statistical method part, the proportional hazards models were carried out; and "assumptions of proportional hazards were visually assessed using Schoenfeld residuals and formally tested by creating interactions with time for all predictors. For covariates violating the proportional hazards assumption (age, BMI and historical job), stratified models were then fitted without the constraint of nonproportionality."

However, I cannot find details of the results based on the description of the steps in the proportional hazards models. For example, what is the results to check assumptions of proportional hazards or what did the Schoenfeld residuals look like; for which analyses the stratified models were performed. More details were needed to be added as supplementary text.

We have now added details in a supplementary file (S1 file). This details which covariates failed the proportional hazards assumption, and test the proportionality of all the predictors in the model by creating interactions with time for the stratified models demonstrating the assumption holds. We think this is a more parsimonious approach than including all the Schoenfeld residual plots (~20 x 3 models).

2) In all association analyses, age, sex, age^2, age^3 among many other confounding covariates were considered. However, age*sex was not considered. From previous literature, age*sex can be an important confounding factor for brain structural and functional metrics. If age*sex is included how much change will be made for the findings?

We agree age*sex interactions have been important confounds in previous studies and these were accounted for in our analyses (age*sex interactions were included within ‘image-related confounds’). Apologies this was not made clear in the previous methods description. We now explicitly state in the methods and figure legends that age*sex was included as a covariate.

3) In Figure 2, is there a significance threshold for the vertex-wise t statistics map? We need to know the significance region.

Thank you for this suggestion. We have added the FDR and Bonferroni T statistics thresholds to Figure 2 legend for better interpretability of the results:

*“False Discovery Rate threshold (3921 tests, T=2.64), Bonferroni threshold (3921 tests, T=4.21).”*

4) Repeated colors were used to represent different categories in Figures 1 and S2. For example, in Figure 1, both the "FIRST" and "rfsMRI" were shown in purple, and "area" and "dMRI" were shown in red. Please change.

RESPONSE: We have amended figures so that there are no repeated colours.

**Reviewer #2:** Topiwala et al. presents a comprehensive analysis to evaluate the association between leucocyte telomere length and multi-modal brain imaging derived phenotypes in 31,661 UK biobank participants. One of the striking finding is that the authors show that LTL has protective effects against dementia. I have following comments.

1. Figure 1: it would be helpful to use triangles to indicate the effect directions (positive or negative).

RESPONSE: Thank you for this suggestion to include a visualization of the effect size in Figure 1 which we agree improves interpretability. Given the large number of partially overlapping data points, we have amended Figure 1 to show the T statistics which made the direction of associations are clearer than triangles (and added Bonferroni and FDR thresholds to show which survive multiple testing correction).

2. It’s hard to follow the paragraph of “Clustered IDPs”. Are these IDP clusters highly correlated with image derived phenotypes, or orthogonal complement to each other? Is each cluster representative of a specific imaging phenotype? How to interpret the edges in networks?

RESPONSE: Apologies this paragraph was not clearly written previously. We have amended the text for clarity. ICA-derived IDP-weight-vectors are statistically independent of each other (by definition) and hence also orthogonal. Subject-weight-vectors are only restricted to being non-co-linear. All clusters are therefore distinct modes of population variation. We have added this explanation to the methods section. Clusters were generally found to be comprised of IDPs from a single imaging modality as explained in the results:

*“In most cases, individual clusters were largely reflecting IDPs from a single imaging modality, with a few exceptions (clusters 6 & 10).”*

We clarify in the methods (‘Image-derived phenotypes’) that ‘edges’ correspond to temporal correlations between nodes’ time series (and anatomical locations of nodes are given in STable 5):

*“…resting-state functional connectivity measures (standard deviations of network nodes timeseries – ‘amplitudes’, as well as temporal correlations between nodes’ timeseries – ‘****edges****’) and task activation.”*

3. In the follow-up study, the authors should exclude other diseases from controls.

RESPONSE: Thank you for this suggestion which we have explored. We repeated the cox proportional hazards models excluding CVA and PD cases from controls in the dementia outcome analyses, excluding PD and dementia in CVA analyses, and excluding CVA and dementia from PD outcomes analyses. There were no appreciable differences to estimates (see table below for comparisons).

4. What’s the associations between neurological and psychiatric disorder phenotypes? (They share strong genetic correlations.)

RESPONSE: A proportion of the dementia cases have Parkinson’s disease (12%) and a proportion have had a previous stroke (11%). This is expected, as both Parkinson’s disease and stroke are recognised causes of dementia (Parkinson’s disease dementia and vascular dementia respectively), and as the reviewer correctly points out there are shared genetic correlations. We have added a comment regarding this to the manuscript:

*“There was a degree of overlap between clinical outcomes (12% of dementia cases had Parkinson’s disease, and 11% had a previous stroke), as expected given Parkinson’s and stroke are known pathways to dementia.”*
